# Exploring a weighted composite score for harmonization of 18F‐MK6240 and 18F‐ Flortaucipir: linearity and correlations with cognitive measures in a head‐to‐head tau PET dataset – The HEAD Study

**DOI:** 10.1002/alz.089551

**Published:** 2025-01-09

**Authors:** Quentin Finn, Guilherme Povala, Guilherme Bauer‐Negrini, Andrea Franco, Tam Vo, Firoza Z Lussier, Livia Amaral, Brian A. Gordon, William E Klunk, Val J. Lowe, Hwamee Oh, Pedro Rosa‐Neto, David N. soleimani‐meigooni, Suzanne L. Baker, Joseph C. Masdeu, Tharick Pascoal, Belen Pascual

**Affiliations:** ^1^ Houston Methodist Research Institute, Houston, TX USA; ^2^ University of Pittsburgh, Pittsburgh, PA USA; ^3^ Department of Radiology, Washington University School of Medicine, Saint Louis, MO USA; ^4^ Mayo Clinic, Rochester, MN USA; ^5^ Brown University, Providence, RI USA; ^6^ McGill University Research Centre for Studies in Aging, Montreal, QC Canada; ^7^ Memory and Aging Center, Weill Institute for Neurosciences, University of California, San Francisco, San Francisco, CA USA; ^8^ Lawrence Berkeley National Laboratory, Berkeley, CA USA

## Abstract

**Background:**

Harmonization of the two most commonly used Tau PET tracers, 18F‐Flortaucipir and 18F‐MK6240 has proven to be complex. Unlike the centiloid scale for amyloid tracers, Tau PET SUVRs of the two tracers are not linearly comparable and vary markedly in dynamic range and sensitivity.

**Method:**

Tau PET SUVRs for Braak stage (1‐6) in 18F‐MK6240 and 18F‐Flortaucipir were obtained from the Longitudinal multicenter head‐to‐head harmonization of tau‐PET tracers (HEAD) project. For training, we used 13 amyloid confirmed AD, 24 amyloid confirmed MCI and 66 amyloid negative controls. To assess linearity and correlations with neuropsychological tests, we included the training data as well as 74 subjects of unknown or inconsistent amyloid status. Two methods of generating outcome measures were used. (1) As a stand‐in for Tau load, similar to the centiloid scale, a score of 100, 50 or 0 was given to participants of the training set by multiplying CDR score by 100. Hierarchical and ridge regression were performed for each tracer using PET‐Braak SUVRs as predictive variables. Regression coefficients were used to calculate an outcome measure for all participants. (2) Principal component analysis (PCA) was applied for each tracer to the vectors of PET‐Braak regional SUVR of the training subset. Projections along the principal axis were calculated for both modalities

**Result:**

Hierarchical, ridge regression and PCA all yielded composite measures with good linear dependence between the two tracers (r^2^ for hierarchical, ridge and PCA were 0.84, 0.80, 0.80, respectively). Regions remaining in the hierarchical regression analyses varied between tracers: predictive regions for 18F‐MK6240 were PET‐Braak 1, 4 and 5, while 18F‐Flortaucipir used only PET‐Braak 1 and 5. Composite measures correlated well with MMSE and total MOCA score with all composite measures r < ‐0.61.

**Conclusion:**

While both tracers may have different sensitivities to regional Tau distributions and disease stages, weighting multiple regions differently for each tracer gives a composite score linearly comparable across tracers that correlates with neuropsychological measures. This study is limited by the low total tau load and impairment level of all participants and would benefit from the inclusion of patients with more disease progression.

